# BCL-G: 20 years of research on a non-typical protein from the BCL-2 family

**DOI:** 10.1038/s41418-023-01158-5

**Published:** 2023-04-08

**Authors:** Mariusz L. Hartman, Malgorzata Czyz

**Affiliations:** Department of Molecular Biology of Cancer, Medical University of Lodz, 6/8 Mazowiecka Street, 92-215 Lodz, Poland

**Keywords:** Prognostic markers, Tumour-suppressor proteins, Cancer genetics, Gene expression, Gene regulation

## Abstract

Proteins from the BCL-2 family control cell survival and apoptosis in health and disease, and regulate apoptosis-unrelated cellular processes. BCL-Gonad (BCL-G, also known as BCL2-like 14) is a non-typical protein of the family as its long isoform (BCL-G_L_) consists of BH2 and BH3 domains without the BH1 motif. BCL-G is predominantly expressed in normal testes and different organs of the gastrointestinal tract. The complexity of regulatory mechanisms of BCL-G expression and post-translational modifications suggests that BCL-G may play distinct roles in different types of cells and disorders. While several genetic alterations of *BCL2L14* have been reported, gene deletions and amplifications prevail, which is also confirmed by the analysis of sequencing data for different types of cancer. Although the studies validating the phenotypic consequences of genetic manipulations of BCL-G are limited, the role of BCL-G in apoptosis has been undermined. Recent studies using gene-perturbation approaches have revealed apoptosis-unrelated functions of BCL-G in intracellular trafficking, immunomodulation, and regulation of the mucin scaffolding network. These studies were, however, limited mainly to the role of BCL-G in the gastrointestinal tract. Therefore, further efforts using state-of-the-art methods and various types of cells are required to find out more about BCL-G activities. Deciphering the isoform-specific functions of BCL-G and the BCL-G interactome may result in the designing of novel therapeutic approaches, in which BCL-G activity will be either imitated using small-molecule BH3 mimetics or inhibited to counteract BCL-G upregulation. This review summarizes two decades of research on BCL-G.

## Facts

- Two validated protein isoforms of BCL-G: BCL-G_L_ and BCL-G_S_ are generated in humans as a result of alternative splicing.
- BCL-G is predominantly expressed in normal testes, and organs of the gastrointestinal tract already at the early stages of fetal development.
- BCL-G level and activity are under the control of multiple proteins, including transcriptional regulators such as p53, PAR bZIP, IRF-1, STAT1, NF-κB and G9a, and a post-translational modifier FAU.
- The involvement of BCL-G in pro-apoptotic activity has been undermined by recent studies in mouse models using genetic manipulation approaches and complementary methods.

## Open questions

- Do validated human isoforms of BCL-G differ in cellular functions?
- Is BCL-G evolutionary conserved and plays similar roles across different species?
- Is the regulation of *BCL2L14* expression and BCL-G activity cell type-specific, and does BCL-G play a considerable role beyond the gastrointestinal tract?
- Could mimicking activity of BCL-G be a therapeutic approach in inflammation-associated colon diseases considering its role in the gastrointestinal tract?

## Introduction

Members of the B-cell lymphoma-2 (BCL-2) family of proteins are involved in the control of cell survival and death in specific physiological and pathological contexts [1]. It has been widely demonstrated and discussed that different BCL-2-like proteins can exert apoptotic and non-apoptotic functions, while they exhibit redundancy in selected cellular processes and cell types [2–9]. The BCL-2 family comprises at least 20 proteins, which can be classified based on apoptosis-related activity and the presence of the structural regions named BCL-2 homology (BH) domains. In general, four BH domains are found in pro-survival proteins, while pro-apoptotic proteins either lack the BH4 domain as shown for BAX, BAK, and BOK, or possess exclusively BH3 domain (BH3-only proteins). Cell fate is determined by complex protein-protein interactions between different members of the family [10–12]. Clinical implications of these interactions have been substantiated with the advent of a novel group of small-molecule agents called BH3 mimetics. BH3 mimetics resemble the activity of specific BH3-only proteins, therefore they induce apoptosis via the inhibition of pro-survival proteins [13–16]. Venetoclax, a first-in-class selective inhibitor of BCL-2 was primarily approved in 2016 by FDA for the treatment of patients with chronic lymphocytic leukemia [17], and then received approval as a therapeutic regimen in other hematological malignancies [18]. In addition, several other selective and dual-targeting BH3 mimetics have demonstrated promising activity in disease, and they undergo extensive investigation. However, the activity of these drugs is (1) restricted to cell types that exhibit dependence on particular BCL-2-like proteins, and (2) limited by adaptive resistance resulting from the application of BH3 mimetics [19–23]. In this respect, unraveling cell dependence on particular pro-survival proteins and deciphering pro-apoptotic BCL-2-like proteins that fuel cell death response, in addition to delineating mechanisms of apoptosis-unrelated functions of BCL-2-like proteins can provide basis for efficient targeting of cancers and non-cancerous disorders. This review summarizes current knowledge on the structure and function of BCL-Gonad (BCL-G), which is an unusual BCL-2-like protein as (1) its long isoform (BCL-G_L_) consists of BH2 and BH3 domains without the BH1 motif, and (2) its role in apoptosis has been undermined by recent studies using genetic manipulation approaches and complementary methods.

## BCL-G: characteristics of gene and corresponding protein

**Fig. 1:**
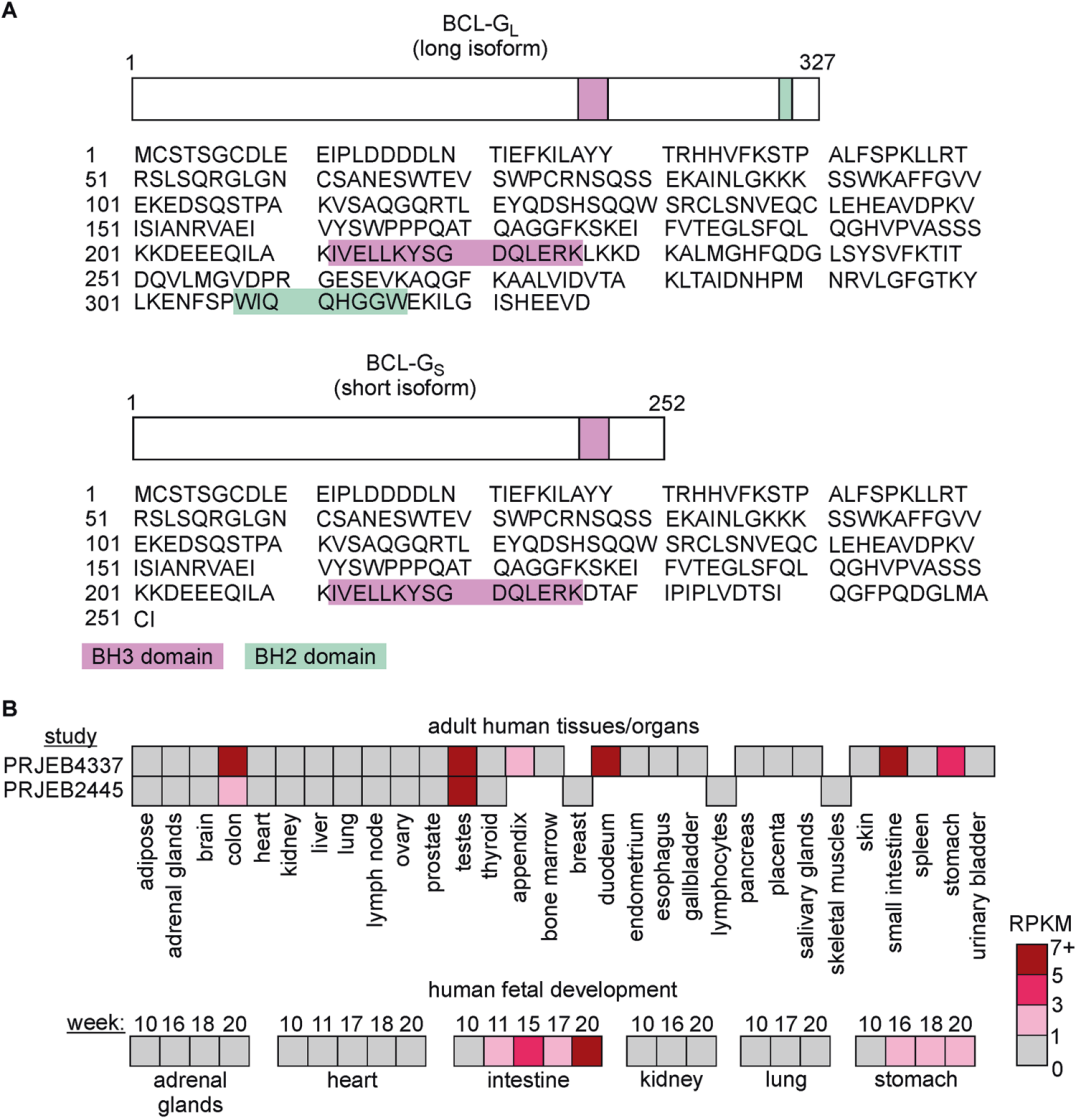
Domain structure of BCL-G and expression of *BCL2L14* in human tissues and organs. **A** Amino acid sequences of human long and short isoforms of BCL-G: BCL-G_L_ and BCL-G_S_, respectively were obtained from *uniprot.org*. The BH3 domain (amino acids from 212 to 226) and BH2 domain (amino acids from 308 to 315) are marked. **B** Upper panel: Transcript levels of *BCL2L14* were assessed by RNA sequencing in different human tissues and organs (BioProject dataset, study PRJEB4337 including samples from 95 individuals representing 27 different tissues, also published [117]; and PRJEB2445 involving 16 tissue samples). Lower panel: Transcript levels of *BCL2L14* were assessed by RNA sequencing in different human fetal tissues between the 10th and 20th weeks of fetal development (BioProject dataset, study PRJNA270632 including 35 human fetal samples from 6 tissues, also published [118]). RPKM reads per kilobase of transcript per million reads mapped.

cDNA of BCL-G (also known as BCL2-like 14) was cloned and initially characterized in Reed’s lab in 2001 [24]. *BCL2L14* encoding BCL-G is located on chromosome 12. As a result of alternative splicing, BCL-G proteins of different lengths can be generated in humans, including short (BCL-G_S_), median (BCL-G_M_), and long (BCL-G_L_) isoforms consisting of 252, 276, and 327 amino acid residues, respectively [24, 25]. It was shown that BCL-G isoforms differed within their C-terminal region. The BH3 domain, which is typical of the vast majority of proteins from the BCL-2 family, was found in all three isoforms, while the presence of the BH2 domain was restricted to BCL-G_L_ [24, 25] (Fig. 1A). Currently available data on Ensembl genome browser indicate that human *BCL2L14* gives rise to 14 transcripts, from which only eight contain an open reading frame (www.ensembl.org). Notably, a splice variant potentially translated into the 276-amino acid isoform of BCL-G is labeled as nonsense-mediated decay (www.ensembl.org), suggesting that BCL-G_M_ protein might not have a biological function. The open reading frame of porcine Bcl-G contains five exons and encodes 329-amino acid protein that demonstrated 71% identity with human BCL-G [26], while in mice, only one isoform resembling human BCL-G_L_ was found [27]. The similarity between murine Bcl-G and human BCL-G_L_ was substantiated by the demonstration that BCL-G_L_ exhibited a very low affinity to BCL-X_L_, while BCL-G_L_ binding to BCL-X_L_ could be enhanced upon deletion of the BH2 domain both in humans and mice [24, 28].

## Expression of *Bcl2l14* in normal tissues

BCL-G_L_ transcript was initially detected in several normal human organs, including the testes, prostate, lung, bone marrow, colon, and pancreas, whereas BCL-G_S_ and BCL-G_M_ were found exclusively in testes [24, 25]. In a more recent study, high levels of both short and long isoforms of BCL-G were found in the human stomach, small intestine, colon, testes, and lymph nodes, but not in the heart and brain, while in the spleen, only BCL-G_L_ was expressed [29]. Therefore, considering BCL-G isoforms separately could be useful to fully delineate their biological functions. In two other BioProject datasets (www.ncbi.nlm.nih.gov/bioproject) involving RNA-seq, a high level of BCL-G mRNA was assessed in a few adult human organs, including the colon, duodenum, small intestine, stomach, appendix, and testes (Fig. 1B, upper panel). Notably, high expression of *BCL2L14* in the gastrointestinal tract was found early during human fetal development, while BCL-G transcript level was low or undetectable in fetal adrenal glands, heart, kidney, and lungs up to 20th week (Fig. 1B, lower panel). Using highly specific monoclonal antibodies, murine Bcl-G was predominantly detected in the small intestine and colon, ciliated epithelial cells in the trachea, bronchi and lungs, CD8+ dendritic cells, bladder, uterus, stratified squamous epithelia of the tongue, salivary and lacrimal glands, and late-stage spermatids of the germinal epithelium [30]. In turn, *Bcl2l14* was not expressed in the interstitial Leydig cells, in cells undergoing earlier stages of spermatogenesis, kidneys, liver, and brain [30]. The transcript level of porcine Bcl-G was assessed at a high level in the heart, lymph nodes, spleen, tonsil, lung, liver, and thymus, while the lowest level was found in the kidney [31].

## Regulation of BCL-G level and activity

### Transcriptional regulation

**Fig. 2:**
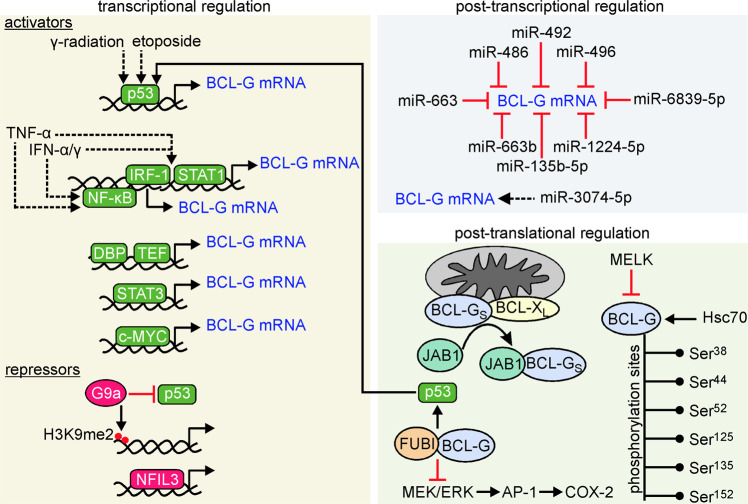
Positive and negative regulators of BCL-G level and activity. In transcriptional regulation, activators are marked in green, while transcriptional repressors are shown in red. For interaction with JAB1, BCL-G_S_ was shown to be relocated from the association with either BCL-X_L_ or BCL-2 [46]. Dotted lines are used to show indirect influence. The phosphorylation sites of human BCL-G were retrieved from www.phosphosite.org.

Several transcriptional regulators of *BCL2L14* expression were identified (Fig. 2). Proline- and acid-rich basic region leucine zipper (PAR bZIP) proteins were shown to control *BCL2L14* expression, particularly the BCL-G_S_ isoform in human embryonic kidney [32], but not in mice [33]. The human *BCL2L14* promoter was especially responsive to activation by the thyrotroph embryonic factor (TEF) in a p53-independent manner, and sensitive to suppression by the nuclear factor interleukin-3-regulated (NFIL3) [32]. In addition, although the *BCL2L14* promoter was also activated by the D-site binding protein (DBP), it was demonstrated that the DBP variant lacking the transactivation domain reduced the formation of active TEF dimers and affected BCL-G_S_ expression [32]. A functional intronic p53-binding site was uncovered in *BCL2L14*, and the BCL-G transcript level increased after tetracycline-inducible activation of p53 [34]. Pharmacological inhibition of G9a, a transcriptional repressor that di-methylates histone 3 lysine 9 (H3K9me2), resulted in striking Bcl-G upregulation via recruitment of p53 to *Bcl2l14*. Notably, this mechanism was not universal for the regulation of all p53-dependent genes [35]. Accordingly, Bcl-G was substantially upregulated in hepatocyte-specific G9a-deficient (*G9a*^*ΔHep*^) mice, and expression of BCL-G and G9a were negatively correlated in the human liver [35]. Knockout of *Wdr5*, encoding a protein involved in chromatin modifications and regulation of mouse embryonic stem cell differentiation, was associated with the downregulation of *Bcl2l14* expression in a p53-dependent manner [36]. p53 might contribute to elevated *Bcl2l14* expression in mice exposed to γ-radiation, whereas high levels of Bcl-G were found in the splenic white pulp, predominantly in cells with fragmented DNA [37]. Other transcriptional regulators of *Bcl2l14* expression such as c-Myc and Stat3 were also considered [37]. On the other hand, no changes in the BCL-G_S_ transcript level and a mild decrease in BCL-G_L_ mRNA abundance were found in normal human colon cell line exposed to nutlin-3, an activator of p53 [38], suggesting cell type-dependent regulation of *BCL2L14* expression by p53.

Interferon regulatory protein 1 (IRF-1)- and signal transducer and activator of transcription 1 (STAT1)-binding sites were also identified in the promoter of *BCL2L14*. In silico analysis of the promoter region revealed interferon regulatory factor element (IRF-E) and interferon-γ (IFN-γ)-activated site (GAS) between −204 and −139, in addition to cAMP responsive element (CRE) that was found downstream [39]. It was demonstrated that co-treatment with IFN-α and IFN-γ substantially increased the mRNA level of BCL-G in human hepatoma cells in both IRF-1- and STAT1-dependent manner [39]. IFN-γ and tumor necrosis factor α (TNF-α) synergistically upregulated short and long isoforms of BCL-G in colonic epithelial cells, while this required STAT1, p65/NF-κB, as well as Brahma (BRM) and Brahma-related gene 1 (BRG1), which are SWI/SNF-associated chromatin proteins [29]. IFN-α2b was also shown to upregulate different BCL-G splice variants in vivo [40].

### Post-transcriptional regulation

Post-transcriptional regulation of BCL-G transcript by different microRNAs (miRs) was demonstrated in different cell types (Fig. 2). BCL-G mRNA was identified as a direct target for miR-663b as miR-663b could complementarily bind within the 3’UTR of BCL-G mRNA in human endometrial cancer cells [41]. BCL-G transcript was targeted by miR-496 in neuroblastoma cells [42], and by miR-486 and miR-663 in fibroblasts and keratinocytes [43]. In turn, it was demonstrated that overexpression of miR-3074-5p was accompanied by a significant increase in BCL-G protein level in human extravillous trophoblast cells [44], although it was not investigated whether this miR was directly associated with BCL-G mRNA, or this was an effect of indirect regulation. miR-135b-5p, miR-492, miR-1224-5p, and miR-6839-5p were found as potentially involved in BCL-G regulation in chondrosarcoma cells [45].

### Post-translational regulation of BCL-G

BCL-G_S_ was shown to interact with JUN activation domain-binding protein 1 (JAB1) both in vitro and in vivo, and JAB1 and BCL-G_S_ co-localized in the cytoplasm [46] (Fig. 2). This interaction could, therefore, affect the intracellular distribution of BCL-G as it was demonstrated that BCL-G_S_ was confined to intracellular organelles [24, 47, 48], whereas BCL-G_L_ was diffused in the cytosol [24]. In addition, BCL-G_S_ bound JAB1 preferentially over BCL-X_L_/BCL-2 when all these proteins were co-expressed [46]. Similar observations were made for porcine BCL-G and JAB1 [31], which is identical to human JAB1 [49].

BCL-G was also identified as a substrate for Ser/Thr maternal embryonic leucine-zipper kinase (MELK), and MELK interacted with BCL-G via the N-terminal moiety of BCL-G [50]. The regulation of Bcl-G by porcine Melk, which exhibits 91% similarity to human MELK, was also shown in swine umbilical vein endothelial cells (SUVECs) [51]. In addition, several putative phosphorylation sites were identified in human BCL-G (www.phosphosite.org) (Fig. 2). The consequences of these modifications and the kinases responsible for them remain, however, to be determined.

Ubiquitin-like modification of BCL-G by monoclonal non-specific suppressor factor β (MNSFβ), known as Finkel-Biskis-Reilly murine sarcoma virus-associated ubiquitously expressed (FAU), was also investigated. *FAU* encodes a fusion protein that consists of the N-terminal ubiquitin-like FUBI (Ubi-L) domain and C-terminal protein S30, whereas the activity of FAU protein is associated with post-translational modification of target proteins by transferring FUBI (Ubi-L) moiety [52]. FUBI (Ubi-L) was shown to covalently bind to Bcl-G through an isopeptide bond between the C-terminal Gly^74^ residue in FUBI (Ubi-L) and Lys^110^ in Bcl-G [27]. Bcl-G-Ubi-L adduct was detected in the spleen, thymus, and brain, but not in the testes [27], indicating that its presence did not overlap with a tissue-specific abundance of Bcl-G. A similar association was demonstrated for porcine Mnsfβ and Bcl-G [53]. The significance of BCL-G modification by FUBI is unlikely to be associated with the formation of polyubiquitin-like chains to promote proteasomal degradation as Lys^48^ is not conserved in ubiquitin-like proteins [52]. Accordingly, either ectopic overexpression or downregulation of FAU did not affect the BCL-G protein level [54]. The association between BCL-G and MNSFβ might have, however, an influence on intracellular signaling. It was demonstrated that Bcl-G-Mnsfβ inhibited mitogen-activated protein kinase (MAPK) pathway in both unstimulated and lipopolysaccharide (LPS)-exposed murine macrophages [55]. It was also shown that co-transfection with Mnsfβ and Bcl-G reduced S-nitrosoglutathione-induced extracellular signal-regulated kinase 1/2 (Erk-1/2) phosphorylation in macrophages [56]. This was accompanied by elevated expression of p53, decreased Cox-2 activity as a result of the downregulation of the activator protein 1 (AP-1) signaling cascade, and apoptosis [56]. In this respect, it can be speculated that a positive feedback loop may exist in the p53-dependent regulation of Bcl-G (Fig. 2). In addition, the contribution of heat shock proteins to the regulation of Bcl-G stability should be considered as it was demonstrated that siRNA-mediated downregulation of Hsc70 was associated with a substantial decrease in the level of Bcl-G protein in mouse macrophages [57].

## Disease-associated genetic alterations of *BCL2L14*

A few types of genetic alterations of *BCL2L14* with specific biological consequences were reported. *BCL2L14*-containing region of chromosome 12 was found commonly deleted in pre-B acute lymphoblastic leukemia (ALL) [58]. In addition, *BCL2L14* was found within the minimal deleted region in acute myeloid leukemia (AML), however, its expression did not differ between AML with and without 12p13 deletion [59]. Allelic losses within chromosome 12 were also reported in patients with prostate cancer [60, 61], oligodontia, and thrombocytopenia [62]. An in-frame insertion of the 33-nucleotide fragment derived from exon 4 of *BCL2L14* corresponding to the median isoform was detected between exon 5 of *TEL* and exon 2 of *AML1*, however, the functional consequences were not determined [63]. *BCL2L14* was identified in a deleted region accompanying translocation leading to ETS variant transcription factor 6 (ETV6)-Runt-related transcription factor 1 (RUNX1) fusion protein in ALL cells [64]. In addition, *BCL2L14-ETV6* fusion gene was mainly present in aggressive triple-negative breast cancer (TNBC) characterized by extensive necrosis, high tumor grade, and mesenchymal phenotype [65]. The frequency of this genetic alteration ranged from 4.4 to 12.2% of TNBC cases, while a fusion between exon 2 of *ETV6* and exon 4 of *BCL2L14* was the most commonly detected [65]. It was also demonstrated that ectopically expressed BCL2L14-ETV6 exerted cytosolic location, and increased cell motility and invasive potential in both TNBC and benign breast epithelial cells. Notably, a product of *BCL2L14-ETV6* rearrangement promoted epithelial-mesenchymal transition and was associated with cell resistance to paclitaxel [65]. As ETV6 is a ubiquitously expressed transcriptional repressor, which was shown to form fusion genes with oncogenic consequences in a number of hematological malignancies and solid tumors [66, 67], it would be crucial to determine whether *BCL2L14-ETV6* fusion occurs in other types of cancer. In addition, a single nucleotide polymorphism variant of *BCL2L14* (rs1544669) in former smokers was associated with a 36% increase in risk for lung cancer [68]. As genetic aberrations of *BCL2L14* rarely involved point mutations [69, 70], publicly available datasets of 310 cancer studies (www.cbioportal.org/) were analyzed with regard to *BCL2L14* genetic alterations (Table 1). The overall frequency of alterations did not exceed 8% in any type of cancer (Table 1). It was accordingly demonstrated that gene amplifications and deep deletions were the most frequent alterations except for non-melanoma skin cancer (Table 1). Therefore, an in-depth investigation of the significance of genetic deletions and amplifications of the part of chromosome 12 containing *BCL2L14* might answer the question about their potential roles in various diseases.

**Table 1:** Ten human cancers with the highest frequency of genetic alterations of *BCL2L14* based on 310 cancer studies available on cBioPortal (www.cbioportal.org/).

		Alteration frequency			
Cancer type	Number of cases	Amplification	Deep deletion	Mutation	Structural variant
Ovarian cancer	1106	7.23%	0.54%	–	–
Germ cell tumor	388	7.47%	–	–	–
B-lymphoblastic leukemia/lymphoma	1026	0.39%	7.02%	–	–
Hodgkin lymphoma	28	7.14%	–	–	–
Lung cancer	39	2.56%	2.56%	–	–
Invasive breast carcinoma	950	2.84%	0.74%	0.21%	–
T-lymphoblastic leukemia/lymphoma	92	–	3.26%	–	–
Breast cancer	5328	1.93%	0.88%	0.02%	0.08%
Skin cancer, non-melanoma	364	–	–	2.75%	–
Pancreatic cancer	2508	1.79%	0.16%	0.16%	–

Only studies reporting combined data on structural variants, mutation and copy number alterations were selected for table preparation.

## Role of BCL-G in normal and diseased cells—is BCL-G pro-apoptotic, pro-survival, or apoptosis-unrelated protein?

Over 20 years of research on BCL-G, a number of studies have reported changes in the levels of BCL-G transcript and/or protein in different biological systems. It was shown that CD3/CD28 stimulation promoted the formation of the Bcl-G-Mnsfβ complex that was accompanied by inhibition of Erk-1/2 and reduced secretion of interleukin-4, as well as apoptosis in Th cells and purified splenic T cells [71]. *BCL2L14* was upregulated in CD4^+^ T cells in patients with systemic lupus erythematosus [72], in the bone marrow of myelodysplasia patients treated with arsenic trioxide and ascorbic acid [73], under hyperbaric air conditions in human diploid embryonic lung fibroblasts [74], and in tongue squamous cell carcinoma cells as a result of hypomethylation of *BCL2L14* [75]. Alterations of DNA methylation patterns to regulate BCL-G levels were also found in human colon adenocarcinoma [76], and lupus [77]. *BCL2L14* was upregulated by nanoparticulate tetraiodothyroacetic acid (tetrac), which reduced viable cell numbers more efficiently than unmodified tetrac in estrogen receptor-negative human breast cancer cells [78, 79]. Upregulation of *BCL2L14* was demonstrated in human osteosarcoma cells exposed to either bilirubin or lithocholic acid, which were shown to have deleterious consequences on osteoblasts, while ursodeoxycholic acid attenuated this effect, suggesting that BCL-G might be involved in osteoporosis in patients with liver diseases [80]. BCL-G mRNA levels also increased in dexamethasone-induced glycogen synthase kinase-3 beta (GSK-3β)-mediated apoptosis in osteoblasts [81]. In a more recent study, it has been demonstrated that BCL-G_L_ was overexpressed in medullary breast carcinoma compared with other subtypes of breast cancer [82]. Thus, the tumor subtype might explain inconsistent observations on either higher or lower levels of BCL-G in breast cancer cells in comparison to normal cells [50, 83]. In turn, lower levels of BCL-G were detected in patient biopsies and cell lines of prostate cancer compared with normal prostate [60, 84], and the transcript level of BCL-G was significantly decreased in human chondrosarcoma cells after iodine-125 (^125^I) seed irradiation [45]. miR-486/miR-663-dependent reduction of BCL-G level accompanied the healing of the thermal injury in the skin [43]. Also, the role of BCL-G in male fertility [85], pregnancy [86], and the correlation between BCL-G level and survival of cancer patients [83, 87] might be considered but require further investigation.

In several studies, RNA interference techniques or overexpression assays were used to determine the phenotypic consequences of direct manipulations of BCL-G level. It was shown that attenuation of *BCL2L14* expression accompanied the development of resistance to neratinib in breast cancer cells [88]. Antisense oligonucleotide-mediated downregulation of Bcl-G was followed by reduced proliferative potential of mitogen-stimulated T cells [27]. In addition, it was shown that suppression of *BCL2L14* protected kidney epithelial cells from detrimental effects of glucose and oxygen deprivation, and limited nephrotoxicity of cisplatin [89], attenuated UV-induced apoptosis in prostate carcinoma and breast cancer cells [83, 84], and prevented apoptosis induced by ectopic *FAU* overexpression in human T-lymphoblastic leukemia cells and embryonic kidney-derived cells [54]. Either downregulation of BCL-G or overexpression of miR-663b, which was involved in suppression of BCL-G, counteracted the influence of pterostilbene, a phenolic compound extracted from the *Vitis sp*., on endometrial cancer cells [41]. More recently, miR-496 was speculated to protect from cerebral ischemia-reperfusion injury via downregulating Bcl-G, while restoration of Bcl-G exhibited more detrimental effects [42]. In addition, BCL-G has been identified during an RNA-seq search for human immunodeficiency virus (HIV) restriction genes involved in response to IFN-α2b [40]. In this respect, BCL-G was markedly upregulated in activated CD4^+^ T cells, while high levels of BCL-G were associated with a decline of HIV RNA in plasma. The capability of BCL-G to diminish the replication of HIV was also confirmed in vitro [40]. Bcl-G was also shown to prevent hepatocarcinogenesis induced by diethylnitrosamine in mice [35]. Mechanistically, Bcl-G was involved in DNA damage-induced apoptosis in hepatocytes following G9a inhibition [35].

All the above-mentioned studies predominantly reported observations accompanying the activity of diverse perturbants and their effects on phenotypic changes rather than provided robust results to exhaustively confirm context-dependent genetic dependence on BCL-G. In this respect, a number of technical and experimental limitations could affect the conclusions [90, 91], and these papers should be mainly treated as a source for additional hypotheses that need to be verified (Table 2). This can be exemplified by the significance of MELK, which was indicated as a potential drug target, but it was shown dispensable for cancer cell viability when CRISPR-mediated *MELK* knockout experimental models were applied [92–96]. For this reason, the biological consequences of the interaction between MELK and BCL-G [50, 51] remain to be determined as it has been demonstrated that *MELK* expression correlated with tumor mitotic activity in one of the studies using *MELK* knockout [95]. Since CRISPR-Cas9 is currently one of the most efficient techniques to generate a complete loss-of-function allele in a gene of interest, is less prone to off-target effects and less affected by gene expression than RNA interference approaches [91], only a few studies have used state-of-the-art genetic manipulations followed by complementary methods to validate the consequences of *BCL2A14* knockout [28–30, 38] (Table 2). Consequently, these studies have consistently excluded the contribution of BCL-G to the regulation of apoptosis [28–30, 38], while a pro-apoptotic function of BCL-G was questioned earlier (reviewed in [97]) despite the presence of exclusively BH3 domain in BCL-G_S_. It was demonstrated that murine Bcl-G might not act as a typical BH3-only protein. Bcl-G did not interact with either Bax or Bak, and could weakly associate with pro-survival proteins from the Bcl-2 family, including Bcl-2, Bcl-X_L_, Mcl-1, Bcl-w, and Bfl-1 [28]. Notably, the deletion of the BH3 domain in Bcl-G did not affect the interactions between Bcl-G and all five pro-survival proteins, which was further evidenced by the replacement of the Bim BH3 domain with Bcl-G-derived BH3 moiety [28]. In addition, *Bcl2l14*-deleted mice had intact gastrointestinal tract [28], including normal architecture and lengths of colon crypts, which was associated with unaffected proliferation and survival of epithelial cells [38]. Importantly, when *Bcl-g*^*−/−*^ mice were grown beyond 1 year, no spontaneous tumor was formed in the entire gastrointestinal tract [38]. Bcl-G was also dispensable for apoptosis in splenic dendritic cells, either in the presence or absence of granulocyte-macrophage colony-stimulating factor [28]. This could suggest that a functional redundancy for Bcl-G could exist as demonstrated for other proteins of the Bcl-2 family [5–7]. However, no changes in the expression of Bcl-2-like proteins were found in the study by Nguyen et al. [38], suggesting that the activity of BCL-G is unlikely to be shared by other proteins from the BCL-2 family at least in adult colonic crypts. Moreover, although an increase in the transcript levels of both BCL-G_L_ and BCL-G_S_ were found during IFN-γ/TNF-α-induced apoptosis in human colorectal cancer cells, downregulation and isoform-specific overexpression of BCL-G revealed that cell death was not dependent on any isoform of BCL-G [29]. In turn, a few apoptosis-unrelated functions of BCL-G were demonstrated. It was shown that murine Bcl-G could be involved in vesicle trafficking and protein transport by interacting with the TRAPP complex, specifically Trappc3, Trappc4, Trappc5, and Trappc6b proteins in intestinal epithelial cells [28]. Any protein of the Bcl-2 family was not found in association with Bcl-G in this experiment employing co-immunoprecipitation [28] that supported the biological function of BCL-G apart from the BCL-2-like proteins. In addition, the biological relevance of the association between the TRAPP complex and BCL-G has been reinforced by a more recent study showing a similar interactome in human cells [98]. It was also demonstrated that Bcl-G regulated the stability of the mucin scaffolding network, and accelerated progression of colitis-associated cancer upon loss of Bcl-G was demonstrated in mice [38]. In this respect, disruption of Bcl-G activity might be linked with colon tumorigenesis [38] as confirmed by a significantly reduced expression of *BCL2L14* in human late-stage colorectal tumors [29, 38]. Also, the depletion of BCL-G affected IFN-γ/TNF-α-induced secretion of inflammatory chemokines CCL5 and CCL20 [29], indicating that BCL-G regulated human gut homeostasis through immunomodulatory activity rather than promoted apoptosis.

**Table 2:** The biological activity of BCL-G demonstrated with distinct strength of evidence in diverse biological systems.

Major observations and findings	Experimental model	Activity of BCL-G	Strength of evidence^a^	Ref.
BCL-G is involved in vesicle trafficking and protein transport via interaction with the TRAPP complex	*Bcl-g*^−/−^ mice; murine intestinal epithelial cells	Apoptosis-unrelated^b^	High (in vivo and ex vivo; *Bcl-g* KO mice)	[28]
BCL-G prevents the progression of colitis-associated cancer via regulation of the mucin scaffolding network	*Bcl-g*^−/−^ mice; murine small intestinal crypts	Apoptosis-unrelated^b^	High (in vivo and ex vivo; *Bcl-g* KO mice)	[38]
BCL-G exerts an immunomodulatory activity via the regulation of secretion of chemokines: CCL5 and CCL20	Intestinal epithelial cells	Apoptosis-unrelated^b^	High (*BCL2L14* KO; induced overexpression of BCL-G; KD—siRNA)	[29]
BCL-G is involved in response to IFN-α2b by diminishing HIV replication	AIDS	Apoptosis-unrelated	Moderate (ex vivo; induced overexpression of BCL-G)	[40]
BCL-G contributes to DNA damage-induced apoptosis after G9a inhibition, and is involved in hepatocarcinogenesis	*G9a*^*ΔHep*^ mice; hepatocytes	Pro-apoptotic	Moderate (in vivo and ex vivo—*G9a*^*ΔHep*^ mice; induced overexpression of BCL-G; KD—shRNA)	[35]
BCL-G_L_ promotes apoptosis in CD4^+^ T cells isolated from patients with systemic lupus erythematosus (SLE)	SLE	Pro-apoptotic	Moderate (ex vivo; induced overexpression of BCL-G; KD—shRNA)	[72]
BCL-G promotes apoptosis accompanying cerebral ischemia-reperfusion (I/R) injury	Neuroblastoma cells	Pro-apoptotic	Moderate (induced overexpression of BCL-G; KD—siRNA)	[42]
BCL-G_L_ enhances basal apoptosis in COS7 cells	Monkey kidney fibroblast-like cells	Pro-apoptotic	Moderate (induced overexpression of BCL-G)	[50]
BCL-G contributes to ultraviolet-induced apoptosis	Breast/prostate cancer and embryonic kidney cells	Pro-apoptotic	Moderate (KD—siRNA)	[54, 83, 84]
BCL-G contributes to detrimental effects of glucose/oxygen deprivation, and nephrotoxicity of cisplatin	Kidney epithelial cells	Pro-apoptotic	Moderate (KD—shRNA)	[89]
BCL-G contributes to pterostilbene-induced apoptosis	Endometrial cancer	Pro-apoptotic	Moderate (KD—siRNA)	[41]
BCL-G downregulation accompanies acquisition of resistance to neratinib	Breast cancer	Pro-apoptotic^c^	Moderate (KD—shRNA)	[88]
BCL-G is upregulated by nano-particulate tetraiodothyroacetic acid	Breast cancer	Pro-apoptotic^c^	Low	[78]
BCL-G is upregulated after exposure to bilirubin or lithocholic acid	Osteosarcoma	Pro-apoptotic^c^	Low	[80]
BCL-G is upregulated during dexamethasone-induced apoptosis	Murine osteoblasts	Pro-apoptotic^c^	Low	[81]
BCL-G downregulation accompanies the healing of the skin	Thermal injury	Not yet determined	Low (ex vivo)	[43]
BCL-G is upregulated in bone marrow of patients treated with arsenic trioxide and ascorbic acid	Myelodysplasia	Not yet determined	Low (ex vivo)	[73]
BCL-G downregulation accompanies reduced activation of T cells	Murine T cells	Not yet determined	Low	[27]
BCL-G downregulation accompanies iodine-125 seed irradiation	Chondrosarcoma	Not yet determined	Low	[45]
BCL-G is upregulated under hyperbaric air conditions	Embryonic lung fibroblasts	Not yet determined	Low	[74]

Unless stated otherwise, the observations and findings were made in vitro and using human cells.

*KD* knockdown, *KO* knockout.

^a^“Low” (observations), “moderate” (observations validated using a single method, or methods with high susceptibility to off-target effects [91]), “high” (solid results obtained using state-of-the-art genetic manipulations and numerous complementary methods [91]).

^b^Pro-apoptotic activity of BCL-G was questioned in experiments performed in parallel.

^c^Expected pro-apoptotic activity of BCL-G requiring extensive experimental confirmation.

## Conclusions and future perspectives

**Fig. 3:**
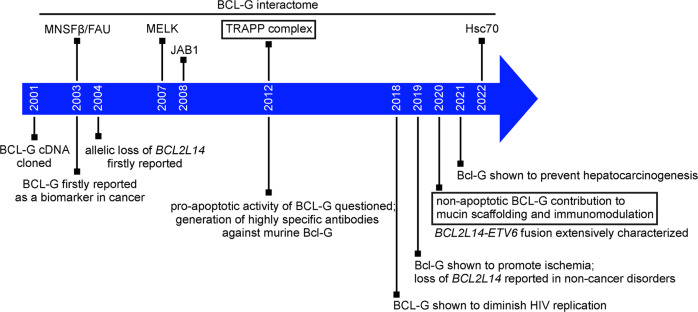
A timeline of major milestones in BCL-G investigation. Discoveries made using state-of-the-art genetic manipulation approaches and complementary methods to validate the role of BCL-G are framed.

In spite of more than two decades of investigation on BCL-G, several crucial milestones (Fig. 3), and recent studies using state-of-the-art genetic techniques that have implied apoptosis-unrelated functions of BCL-G in the gastrointestinal tract (Table 2), the biological role of BCL-G remains to be fully defined. Further efforts focusing on various types of cells are required to find out more about the cell type- and context-specific activities of BCL-G. It also needs to be determined how conserved BCL-G is and whether it plays similar or different functions across species. Although the phylogenetic tree of BCL-2 homologous proteins, rooted with CED-9 sequences from *Caenorhabditis* and including BCL-G, has been demonstrated [99], BCL-G remains understudied in this respect. In general, the phylogenetic analyses have revealed quite similar structures and mechanisms of interactions between BCL-2-like proteins despite their either convergent or divergent history of evolution [100–107]. In addition, as alternatively spliced variants lacking certain motifs are found in the family as exemplified by BCL-X_S_ and BCL-G_S_, it has been speculated that some BCL-2-like proteins derive from a common ancestry with multidomain members through gene duplication and exon loss [108–110]. Therefore, it will be crucial to delineate whether BCL-G_S_ and BCL-G_L_ resemble two isoforms of BCL-X, BCL-X_S_ and BCL-X_L_ exerting diverse activity [111]. In contrast to BCL-X_L_, however, the BCL-G_L_ isoform contains BH2 and BH3 domains without the BH1 domain, which is not typical for proteins of the BCL-2 family. In this respect, BCL-2 family Kin (BFK) is another example of a BCL-2-like protein, which is structurally similar to BCL-G_L_ [112]. A recently published report on BFK might shed light on the directions of further research on BCL-G [113]. Biophysical interaction analysis revealed that full-length BFK did not interact with other BCL-2-like proteins, but instead it was functionally reminiscent of BID, in which the BH3 domain was released upon caspase-mediated cleavage and truncated form (tBID) underwent a conformational alteration [113]. In addition, as protein modifications with ubiquitin-like moieties were shown to regulate the activity of target proteins, their intracellular localization and interactions with other proteins [114], the activity of FAU via BCL-G should be taken into account when the role of BCL-G is identified. The interaction of BCL-G with FAU is also likely to be of clinical significance as decreased FAU levels related to unfavorable outcomes were found in a number of tumors, including prostate, breast, and ovarian cancer [83, 84, 115]. The *FAU* polymorphic variant (rs769440) has been recently associated with recurrent pregnancy loss [116]. Therefore, understanding the BCL-G interactome may facilitate the design of novel therapies, in which the activity of BCL-G will be either imitated using small-molecule BH3 mimetics or inhibited to the attenuate effects of BCL-G upregulation. It will be also valuable to assess BCL-G levels under clinically-relevant scenarios. In this respect, G9a expression, and the correlation between G9a and clinical outcomes will be evaluated in patients with vulvar cancer (recruiting study NCT03695809). For now, BCL-G remains a protein with an apoptosis-unrelated role in intracellular trafficking, immunomodulatory functions and regulation of the mucin scaffolding network within the gastrointestinal tract.

## Data Availability

This review article does not present any new primary data. References for publicly available datasets concerning gene expression, genetic alterations and protein structure are given in figure and table legends.
